# Transforming Growth Factor-Beta (TGFβ) Signaling Pathway in Cholangiocarcinoma

**DOI:** 10.3390/cells8090960

**Published:** 2019-08-23

**Authors:** Panagiotis Papoutsoglou, Corentin Louis, Cédric Coulouarn

**Affiliations:** Inserm, Univ Rennes, Inra, Institut NuMeCan (Nutrition Metabolisms and Cancer), UMR_S 1241, 35033 Rennes, France

**Keywords:** TGFβ, cholangiocarcinoma, liver cancer, signaling, noncoding RNA

## Abstract

Cholangiocarcinoma is a deadly cancer worldwide, associated with a poor prognosis and limited therapeutic options. Although cholangiocarcinoma accounts for less than 15% of liver primary cancer, its silent nature restricts early diagnosis and prevents efficient treatment. Therefore, it is of clinical relevance to better understand the molecular basis of cholangiocarcinoma, including the signaling pathways that contribute to tumor onset and progression. In this review, we discuss the genetic, molecular, and environmental factors that promote cholangiocarcinoma, emphasizing the role of the transforming growth factor β (TGFβ) signaling pathway in the progression of this cancer. We provide an overview of the physiological functions of TGFβ signaling in preserving liver homeostasis and describe how advanced cholangiocarcinoma benefits from the tumor-promoting effects of TGFβ. Moreover, we report the importance of noncoding RNAs as effector molecules downstream of TGFβ during cholangiocarcinoma progression, and conclude by highlighting the need for identifying novel and clinically relevant biomarkers for a better management of patients with cholangiocarcinoma.

## 1. Cholangiocarcinoma

### 1.1. Epidemiology and Risk Factors

Cholangiocarcinomas (CCAs) comprise heterogeneous hepatobiliary tumors with cholangiocyte differentiation features. CCA is the second most common hepatic malignant tumor after hepatocellular carcinoma (HCC). CCAs are classified as intrahepatic (iCCA), perihilar (pCCA), and distal (dCCA), according to their anatomic location [1]. CCA subtypes differ by their epidemiology, etiology, pathogenesis, and thus, clinical management and targeted therapeutic options. Although challenges in the classification of CCA made it difficult to quantify, a gradual increase in CCA incidence was reported worldwide during the last few decades [2]. The discrepancy in incidence of sporadic CCA worldwide is associated with well-established risk factors. Higher incidence of CCAs in Eastern countries reflects a geographical disparity in the prevalence of risk factors [1]. For instance, hepatobiliary flukes such as *Clonorchis sinensis* and *Opisthorchis viverrini* are both common risk factors in Southeast Asia, where CCA is recognized as a non-rare cancer [3,4]. Hepatitis B and C have also been identified as risk factors for CCAs, especially for iCCA. While hepatitis C is prevalent in Western countries, hepatitis B is strongly associated with CCAs in Asia [5]. Furthermore, the association between primary sclerosing cholangitis (PSC) and CCA is well-established. Studies from Western countries reported that PSC patients developed CCAs with a prevalence ranging from 5% to 15% and a yearly developmental rate of CCAs ranging from 0.5% to 1.5% [6]. Other risk factors including hepatholithiasis, metabolic syndrome, alcohol, smoking, and diabetes are also suspected to be involved in CCA onset, all of these factors contributing to generate a pro-inflammatory environment in biliary tracts [5,7]. As a result of its silent nature, CCA is generally diagnosed at the advanced stage, when therapeutic options are limited. Surgery is currently the best option for CCA treatment, even though tumor size, metastasis, and lymph node invasion make it unfeasible in more than 65% cases [8]. For the resectable early stage CCAs, survival at five years ranges from 15% to 40%, but is associated with a high risk of recurrence [9]. However, for unresectable advanced CCAs, overall survival (OS) is below 15 months [9]. The lack of a clear and global picture of cellular and molecular alterations, which occur in aggressive CCAs, might account for this unfavorable clinical outcome. Improvement in CCA outcome relies on efforts toward a better understanding of cholangiocarcinogenesis mechanisms to develop efficient targeted therapies, as well as the identification of reliable biomarkers for early detection.

### 1.2. Molecular Pathogenesis

CCA is frequently associated with drastic changes in the tumor microenvironment, including intense extracellular matrix remodeling and inflammation, which modulate the activity of signaling pathways involved in tumor onset and progression [10,11]. These alterations lead to an aberrant expression and/or activation of key cytokines, tyrosine kinases, and ultimately transcription factors which control cell fate [12,13,14]. As an example, an increase of interleukin 6 (IL6) secretion by CCAs and desmoplastic stromal cells lead to the activation of STAT3, a latent cytoplasmic transcription factor. IL6 binds to the dimerized GP130 receptors associated with Janus family kinases (JAK) including JAK1, JAK2, and TYK2, leading to STAT3 phosphorylation and activation [15]. STAT3 acts not only as an activator of transcription but also as a signal transducer. Its activation modulates a variety of genes involved in cell survival, proliferation, and migration. An elevated expression of STAT3 in CCA tumor tissues has been identified as an independent prognostic factor for OS and disease-free survival (DFS) [16]. There is also evidence demonstrating that epidermal growth factor receptor (EGFR) contributes to CCA progression by disturbing cell–cell adhesion and cell motility, triggering epithelial to mesenchymal transition (EMT) and thus promoting a pro-metastatic process [17]. EGFR activation by its ligands (e.g., EGF, TGFA, AREG) initiates several signal transduction cascades, including extracellular signal-regulated kinases (ERK) 1/2 and serine/threonine kinase 1 (AKT1), which are both implicated in cell proliferation and migration [18].

Developmental pathways are well-conserved axes required for biliary tract cell differentiation and proliferation. Unsurprisingly, dysregulations of these pathways have been described in CCAs. Recent evidence showed that a persistent activation of Notch signaling is associated with iCCA [19]. A study using a mouse model of iCCA revealed that the Notch axis was critical in hepatocyte conversion into biliary lineage, and therefore, an enhanced activity of this pathway may contribute to malignant conversion of hepatocytes into CCAs [20]. Hedgehog (HH) is another developmental pathway involved in critical cell fate decision, including apoptosis, stem cell maintenance, and wound healing [21]. HH pathway was identified as a key player in tumor initiation in several cancers, including CCAs [22,23]. El Khatib et al. investigated the effects of blocking the HH pathway using cyclopamine in vitro on human CCA cells, and in vivo using xenograft of CCA cells in mice. Cyclopamine is a steroidal alkaloid isolated from *Veratrum californicum*. It plays a critical role in embryonic development by hindering the HH pathway. Such a muting of the HH pathway resulted in a blockage of CCA cell migration and invasion. In CCA xenografts, cyclopamine treatment led to a significant inhibition of tumor growth, highlighting the importance of the HH pathway in CCAs and the clinical relevance of its inhibition [24].

High throughput strategies identified numerous genetic, epigenetic, and genomic alterations in CCA, and highlighted specific targetable signaling pathways. Among them, mutations in isocitrate dehydrogenases genes (*IDH1* and *IDH2*) and chromatin-remodeling genes, including AT-rich interaction domain 1A (*ARID1A*), as well as gene fusions involving fibroblast growth factor receptor 2 (*FGFR2*) were frequently detected in CCAs [25,26,27,28]. Mutations of the tumor suppressor *TP53* are also commonly found in CCAs (44% of cases), so are mutations of *KRAS* (17% of cases) and *SMAD4* (17% of cases) [29]. According to the same study, seven genes (*TP53*, *SMAD4*, *KRAS*, *RNF43*, *NDC80*, *ROBO2*, and *GNAS*) scored as the top mutated genes in *Opisthorchis viverrini*-associated CCAs, with false discovery rates ranging from 2.1 × 10^−5^ to 0.29. More recently, protein tyrosine phosphatase non-receptor 3 (*PTPN3*) was reported to be frequently mutated in iCCA and associated with tumor recurrence [30].

### 1.3. Targeted Therapies

Although significant progress in understanding the molecular basis of CCA pathogenesis has been achieved, there is no approved molecular targeted therapy that significantly improves patient survival. Thus, there is a critical need of designing innovative therapeutic strategies and biomarkers for a better management of patients with CCA, which remains a deadly cancer with a very poor prognosis [9]. Previous genomic characterizations of the stroma in iCCA highlighted clinically relevant biomarkers predictive of patient survival, some of them being related to the transforming growth factor β (TGFβ) signaling [11,31,32]. Several clinical trials are still in progress based on genetic alterations observed in CCAs. For example, the therapeutic effect of the multi-targeted tyrosine kinase inhibitor, ponatinib, has been tested in two patients with advanced CCA tumors associated with activating *FGFR2* gene fusions. This trial resulted in an efficient anti-tumor response characterized notably by shrinkage of metastatic lymph nodes [33]. Promising results using IDH1 and IDH2 inhibitors have been also reported. Two mutant IDH inhibitors, enasidenib (AG-221) and ivosidenib (AG-120), have been approved in ongoing trials on Acute Myeloid Leukemia (AML), and their therapeutic benefits are now studied in other malignancies including CCA [34].

## 2. Transforming Growth Factor Beta (TGFβ) Pathway

### 2.1. TGFβ Signaling: From Receptor Activation to Transcription Of Target Genes

**Figure 1:**
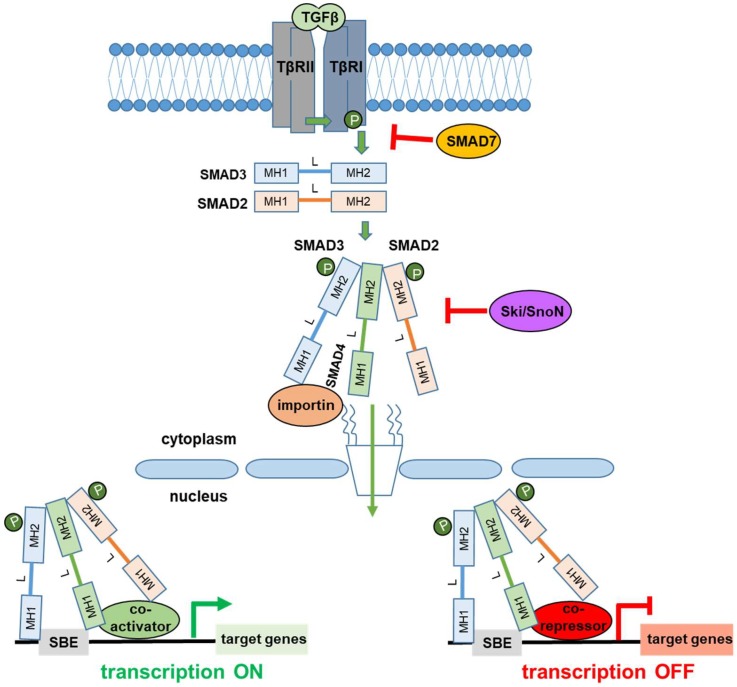
Basics of transforming growth factor β (TGFβ) signaling. Binding of TGFβ ligands to their receptors initiates the signal, through the phosphorylation of TβRI by TβRII. Then, TβRI transmits the signal to SMAD2 and SMAD3 by phosphorylating their MH2 domains. This phosphorylation enables SMAD2 and SMAD3 activation and the formation of a complex with SMAD4, which, in turn, enters into the nucleus through nuclear pores with the assistance of importins. In the nucleus, the activated SMAD complex regulates the expression of target genes, in a positive or negative manner, depending on its association with transcriptional coactivators or corepressors. The signaling is subjected to negative regulation by SMAD7, which prevents SMAD2/3 activation and induces degradation of TβRI, and by Ski/SnoN, which interferes with the formation of active SMAD2/3/4 complex. MH1: MAD homology 1 domain, L: Linker domain, MH2: MAD homology 2 domain, TβRI: type I TGFβ receptor, TβRII: type II TGFβ receptor.

Members of the TGFβ family are pleiotropic cytokines that exhibit important roles in tissue homeostasis, cell differentiation, and embryonic development. Extracellular TGFβ ligands bind to transmembrane type I and type II TGFβ receptors (TβRI and TβRII, respectively), thereby initiating a signaling cascade that ultimately leads to altered expression of protein-coding and noncoding target genes [35,36]. Initiation of the TGFβ pathway involves binding of ligands to TβRII, formation of a heterotetrameric complex between TβRII and TβRI, and phosphorylation of TβRI by TβRII, the latter possessing serine/threonine kinase activity in its intracellular domain. Phosphorylation of TβRI turns on its serine/threonine kinase activity, resulting in the phosphorylation of the members of the SMAD family, SMAD2 and SMAD3, which interact, at their carboxy-terminal domain, with the common mediator SMAD4 and transduce the signal into the nucleus. The transport of the activated SMAD complex into the nucleus is mediated by proteins of the nuclear pore, such as importin-β1 and CAN/Nup214, and requires the GTPase RAN [37]. In the nucleus, the SMAD complexes associate with various coactivator or corepressor factors to regulate gene transcription in a positive or negative manner, respectively (Figure 1).

Structurally, the transcription factors SMAD2, SMAD3, and SMAD4 consist of three main domains, an N-terminal (or MH1), a central (or linker), and a C-terminal (or MH2) domain [38]. The MH1 domain enables SMAD3 and SMAD4 to directly recognize and bind to specific SMAD-binding elements (SBEs) at promoter or enhancer DNA regions [39]. The linker domain is subjected to extensive post-translational modifications that influence stability and interactions with proteins from other signaling pathways. The MH2 domain is important for the interaction of SMAD2 and SMAD3 with TβRI, SMAD4, and other protein partners.

In parallel to TGFβ-induced SMAD activation, TGFβ receptors can also induce non-SMAD pathways, such as MAP kinase (MAPK), phosphatidyl-inositol-3′ kinase (PI3K,) and the cell polarity regulator PAR6, which can mediate its biological functions [40]. The various physiological actions of TGFβ signaling require control mechanisms to ensure that the magnitude of the signal meets the temporal, spatial, or developmental needs of the cellular systems. Thus, several mechanisms prevent an overactivation of the TGFβ signaling. Notably, SMAD7 and Sloan Kettering Institute proto-oncogene (SKI)/SKI-related novel gene (SnoN) proteins are the best described negative regulators of TGFβ signaling. SMAD7 is a member of the SMAD family and its inhibitory role on TGFβ signaling is exerted in two ways. First, SMAD7 antagonizes with SMAD2/3 for binding to TβRI receptors, thereby limiting the phosphorylation of SMAD2/3. Second, SMAD7 promotes the ubiquitin-dependent proteosomal degradation of TβRI receptor by recruiting E3-ubiquitin ligases (SMURFs) to the receptor [41]. SKI and SnoN proteins interact with SMAD2/3 or SMAD4 and interfere with the formation of an active SMAD2/3-SMAD4 complex, thus also interfering with the propagation of the TGFβ signal [41] (Figure 1).

### 2.2. Crosstalk of TGFβ Signaling with Other Signaling Pathways

TGFβ-mediated cellular responses are frequently achieved by the cooperation of TGFβ with other signaling pathways. Thus, the crosstalk between TGFβ and a plethora of pathways, such as WNT, HIPPO, NF-κB, Notch, hedgehog, JAK/STAT, MAPK, and PI3K-AKT, is supported by the current literature [42]. Interestingly, most of these pathways are deregulated in CCAs [43]. As an example, TGFβ and HIPPO pathways are functionally associated to regulate CCA progression. Hyperactivation of yes-associated protein-1 (Yap1) and transcriptional coactivator with PDZ-binding motif (Taz), due to a genetic depletion of Mps One Binder kinase activator (Mob)1a/1b in mouse liver, results in an increased incidence of combined HCC-CCA and iCCA. These effects are accompanied by increased levels of TGFβ2 and TGFβ3 ligands. Interestingly, a positive correlation between YAP1 and SMAD2 activation has been shown in patients with HCC-CCA and iCCA [44]. Interleukin-6 (IL-6) and TGFβ pathways also converge to potentiate malignancy and resistance to chemotherapy in biliary tract cancer [45].

### 2.3. Physiological Responses in the Liver

TGFβ signaling dictates transcriptional programs, which influence diverse physiological processes, such as cell cycle arrest, apoptosis, EMT, and immune surveillance. When the tight regulatory mechanisms of TGFβ signaling activity are circumvented, pathological conditions, such as fibrotic diseases and tumorigenesis, may arise due to excessive and uncontrolled activity of the pathway. For example, the pathogenesis of liver fibrosis involves extravagant production and deposition of extracellular matrix (ECM) components i.e., collagen, produced at the liver tissue, in response to chronic liver damage [46]. At the onset of liver fibrosis, a combinatorial action of inflammatory responses, infiltrating immune cells, and cytokine signaling (e.g., TGFβ) triggers the activation of hepatic stellate cells (HSC) and their transition towards myofibroblasts [47]. Failure of recovery, as a consequence of constant liver injury, favors a persistent pro-fibrotic microenvironment, ultimately leading to severe fibrotic disease. Both SMAD-dependent and non-SMAD signaling can mediate the effects of TGFβ in HSC, such as the induction of connective tissue growth factor (CTGF) [48].

In epithelial cells, TGFβ blocks cell proliferation, mainly through the induction of cyclin-dependent kinase (CDK) inhibitors, such as P21, P15, and P27, and the reduction of the oncogene MYC [49]. The pro-apoptotic effects of TGFβ are well-described in normal liver or HCC cell lines. For example, TGFβ induces apoptosis of Hep3B HCC cell line, a process that involves the activation of caspases. In the case of HUH7 cells, TGFβ-mediated apoptosis engages the activation of caspases and the downregulation of anti-apoptotic proteins, such as BCL-XL and XIAP [50]. In other cellular models, the involvement of the c-Jun N-terminal kinase (JNK) pathway [51], the induction of the tumor suppressor TGFβ inducible early growth response protein 1 (TIEG1) [52], or the death-associated protein kinase (DAPK) [53] have been reported as mediators of apoptosis downstream of TGFβ.

TGFβ also plays a central role in promoting EMT, a process whereby epithelial cells lose their well-defined morphology and adhesion and acquire mesenchymal traits, allowing them to migrate from their original site [54]. EMT is crucial for normal embryogenesis, however, during tumor progression, epithelial carcinomas can profit from hyperactivation of EMT-inducing signaling pathways, such as WNT and TGFβ, to gain migratory properties, enabling them to metastasize. Many reports pinpoint the positive contribution of EMT to metastasis [55], although a few of them support that EMT is not a determinant factor for pancreatic [56] or lung metastasis [57], but is associated with increased resistance of these cancers to chemotherapeutic agents. In fact, EMT has been also linked to the generation of cancer stem cells (CSCs), defined as undifferentiated cancer cells, which possess stem cell characteristics, contribute to tumor heterogeneity, and show low sensitivity to chemotherapy [58]. Therefore, by positively regulating EMT, TGFβ is also capable of conferring stemness features to cancer cells, as described in HCC [59]. At the molecular level, TGFβ restricts epithelial phenotypes either by downregulating components of tight junctions, such as E-cadherin, or by redistributing them away from the cell membrane, thereby leading to their disassembly. In addition, TGFβ induces the expression of EMT transcription factors (SNAIL, SLUG, ZEB1, ZEB2, TWIST) and mesenchymal markers (fibronectin, vimentin, collagens) to achieve EMT [60].

Immune system responses are also controlled by the TGFβ pathway, which, in general, exhibits immune-suppressive effects. Immune suppression by TGFβ is a mechanism by which cancer cells may escape immune surveillance. TGFβ disrupts the ability of immune cells to recognize and eliminate cancer cells by shifting the differentiation of naïve CD4+ cells towards T-regulatory cells, thereby restricting the production of effector T cells. In addition, it prevents natural killer cells from destroying tumor cells. A characteristic example for the role of TGFβ in cancer progression, via modulation of the immune system, derives from a study using CCA cells. The findings of this study suggested that interference of the TGFβ pathway by neutralizing antibodies against TβRII in dendritic cells (DC) caused increased activation of effector T cells and, in turn, enhanced targeting and lysis of co-cultured CCA cells by the immune system [61]. In addition, another cause of CCA progression is chronic inflammation, in response to injury or due to the presence of high levels of pro-inflammatory cytokines at the bile duct [62]. Interestingly, TGFβ induces the expression of IL6 in iCCA cell lines, an effect that facilitates CCA growth [63]. Other major contributors of tumor progression are the tumor associated macrophages (TAM) [64]. These immune cells are recruited at the tumor site and secrete cytokines, such as TGFβ, thereby creating a pro-inflammatory microenvironment that favors cancer progression. Also, TAM and other immune cells have been identified at tumor areas in patients with eCCA and are correlated with poor prognosis [65]. Interestingly, addition of supernatant from HuCCT1 CCA cells on TAM enforced the latter to express high levels of TGFβ, IL10, and VEGF, suggesting that the interplay between TGFβ and TAM is of high importance in CCA tumor microenvironment [66].

### 2.4. TGFβ Functional Duality in Cancer

TGFβ is a challenging target for cancer treatment, due to its ability to both inhibit and facilitate tumor progression. During tumor initiation, TGFβ exhibits tumor-suppressing functions by halting proliferation and inducing programmed cell death. In contrast, in advanced malignancies, TGFβ preferentially exerts tumor-promoting actions by affecting the behavior of the cancer cells themselves, or by creating a favorable microenvironment for tumor growth [67]. The unresponsiveness of advanced cancers to the tumor-restricting properties of TGFβ is a consequence of either genetic mutations of downstream cytostatic genes that, otherwise, are induced by the pathway, or mutations in components of the core signaling pathway, such as *TGFBR2* and *SMAD4* [68]. For example, CCA cells do not respond to the growth inhibitory effects of TGFβ, due to high expression levels of cyclin D1. On the other hand, normal biliary epithelial cells, expressing physiological levels of cyclin D1, undergo cell cycle arrest in response to TGFβ [69]. In the case of liver cancer, TGFβ induces cytostatic and pro-apoptotic factors at early stages of cancer, but later on it promotes EMT and also stimulates the generation of cancer associated fibroblasts (CAF) in the tumor stroma, which maintain active TGFβ signaling and contribute to metastasis [70].

## 3. TGFβ Pathway in Cholangiocarcinoma

### 3.1. Genomic Alterations

In many cancers, genes encoding members of the TGFβ pathway are frequently subject to mutations, reflecting the importance of this pathway in tumor progression [71]. Indeed, studies involving whole genome or exome sequencing from human CCA tissues have recorded genomic aberrations in main signaling pathways, and among them, TGFβ has a special place [27,28]. Using a large cohort of 103 iCCA patients, Zou and coworkers identified RAS/PI3K, P53, and TGFβ pathways to be influenced by alterations in the exome. *SMAD4* was described as one of the 25 significantly mutated genes, with a mutational rate of 4% (*P* < 0.01) [28]. In another study, *SMAD4* was mutated in 3.6% of patients with iCCA (n = 55) and in 25% of patients with eCCA (n = 20) [72]. Although, this difference in the frequency of *SMAD4* mutations between iCCA and eCCA was not statistically significant (*P* = 0.333), probably as a result of a limited number of eCCA cases, it suggests that eCCA exhibits a molecular phenotype that resembles pancreatic cancer rather than iCCA [29,72]. The expression of SMAD4 was also evaluated by immunohistochemistry in normal liver and iCCA tissues with different differentiation status and clinical stages. SMAD4 inactivation was found in 22 out of 49 iCCA specimens (44.9%, *P* = 0.029), whereas all normal liver tissues (n = 9) expressed SMAD4. A significant negative correlation between SMAD4 expression and advanced clinical stages was highlighted [73]. Indeed, loss of SMAD4 expression was associated with lymph node and intrahepatic metastasis (*P* < 0.001), poorly to moderately differentiated histological grade (*P* = 0.013), and advanced TNM stage (*P* = 0.018). In addition, the expression of SMAD4 and cell cycle regulators (P53, P16, P27, cyclin D1, and Rb) was measured in 42 resected iCCAs by immunohistochemistry. Loss of expression of SMAD4 was reported in 45.2% of cases, as well as genes encoding cell cycle inhibitors (e.g., 35.7% for P16) [74]. Loss of SMAD4 was positively correlated with advanced pTNM stage (*P* = 0.039). Taken together, these studies suggest that different mechanisms contribute to inactivate SMAD4 and possibly the tumor suppressive arm of the TGFβ pathway, both in eCCA, involving *SMAD4* inactivating point mutations, and in iCCA, involving a transcriptional down-regulation of SMAD4.

### 3.2. TGFβ Regulates CCA Tumor Progression

TGFβ is one of the main signaling pathways that promotes CCA progression. Many studies support the TGFβ-mediated induction of EMT in CCA cell lines [75]. Stimulation of human CCKS-1 and TFK-1 cells with TGFβ led to a significant induction of SNAIL, VIM, and S100A4 and the reduction of E-cadherin and cytokeratin 19, thereby promoting migration and invasion [76]. TGFβ also exhibits pro-EMT functions in iCCA cell lines KKU-M213 and HuCCA-1. Enhanced expression of VIM and SLUG and secretion of the metalloproteinase MMP9, accompanied by a concomitant increase in cell migration and invasion, were observed after TGFβ treatment [77]. TGFβ stimulation activated both SMAD-dependent and SMAD-independent pathways, exemplified by the induced phosphorylation of SMAD2/3 and ERK1/2, respectively. Interestingly, inhibition of the kinase activity of MEK diminished the induction of EMT but, in contrast, potentiated the anti-proliferative effects of TGFβ [77]. This observation raises the possibility that selective blockade of the noncanonical TGFβ signaling may suppress the pro-tumorigenic, while preserving the anti-tumorigenic effects of TGFβ in CCA. Disruption of TGFβ pathway in human CCA primary cell cultures, using the TβRI kinase inhibitor LY2157299 (galunisertib), compromised cell migration. Notably, the same inhibitor did not influence cell cycle progression or apoptosis, indicating a selective inhibitory effect towards EMT [78]. Moreover, inhibition of the kinase CK2, which is linked to TGFβ signaling, attenuated proliferation and increased apoptosis of the primary CCA cells [78]. The observed effects propose a combinatorial use of TβRI and CK2 kinase inhibitors for the treatment of CCAs. In another report, treatment of Huh28 and RBE CCA cell lines with the 3-hydroxy-3-methylglutaryl-coenzyme-CoA (HMG-CoA) reductase inhibitor, lovastatin, resulted in reduced TGFβ1 expression as well as reduced tumor cell proliferation and migration [79]. An interesting link between TGFβ signaling, EMT, and the generation of CCA cells with stemness properties was provided by Shuang and co-workers [80]. According to this study, exposure of TFK-1 cells to TGFβ resulted in the acquisition of a mesenchymal phenotype and an increase in the population of cells positive for the cancer stem cell marker aldehyde dehydrogenase1 (ALDH1). Moreover, decreased cell death rates of TFK-1 cells in response to the DNA-damaging agent 5-fluorouracile (5-FU) was observed as a consequence of TGFβ treatment, suggesting that TGFβ confers chemoresistance of CCA cells to anti-cancer drugs [80]. It is worth noting that BMP7, a ligand that belongs to the TGFβ family of proteins but signals through a different combination of receptors and effector SMADs, is capable of antagonizing the effects of TGFβ-induced EMT in CCAs. Indeed, combinatorial treatment of M213 and M139 CCA cells with TGFβ1 and BMP7 attenuated migration and the increased expression of the EMT markers TWIST and N-cadherin, observed after TGFβ1 stimulation [81].

The effects of hepatitis B (HBV) and C (HCV) viruses on the progression of iCCA were evaluated in vivo using zebrafish as an experimental model. Livers from animals overexpressing both hepatitis B virus X (HBx) and hepatitis C virus core (HCP) proteins developed fibrosis and iCCA characterized by activated noncanonical TGFβ signaling, exemplified by enhanced MAPK and SMAD linker phosphorylation. In addition, in vivo disruption of TGFβ1 expression by morpholinos attenuated fibrosis and iCCA progression, suggesting the importance of TGFβ signaling during hepatitis-induced iCCA [82]. Moreover, experiments in transgenic zebrafish, whereby the expression of *tgfb1* was specifically induced in hepatocytes, showed increased incidence of HCC and CCA as a consequence of *tgfb1* chronic expression. Interestingly, HCC tumors were characterized by a switch from Smad-dependent to Erk-dependent TGFβ pathway, whereas CCA tumors exhibited activation of both canonical and Erk pathways [83].

On the other hand, there are very few reports about a tumor-suppressing role of the TGFβ pathway in CCAs. TGFβ-treated RBE human CCA cells exhibited elevated apoptosis, a process that was SMAD-dependent and augmented by inhibition of the c-Jun N-terminal kinase (JNK), using the chemical inhibitor SP600125 [84]. Blocking JNK activity not only resulted in enhanced TGFβ-induced apoptosis, but also in increased levels of C-terminal phosphorylated SMAD2 and SMAD3 and a general induction of TGFβ-dependent transcriptional responses. Nevertheless, JNK inhibition did not influence SMAD linker phosphorylation, implying alternative molecular mechanisms for the inhibitory role of JNK on SMAD activation, in this context [84]. In a recent report, PTPN3, a protein tyrosine phosphatase which acts as a tumor suppressor, was shown to enhance TβRI stability, independently of its catalytic activity. PTPN3 was described as an antagonist of SMURF2 by overlapping with its binding domain to TβRI. SMURF2 is a well-known ubiquitin E3 ligase, recruited by SMAD7 and targeting TβRI for proteasomal degradation [85]. Interestingly, *PTPN3* point mutations (L232R) may disrupt its interaction with TβRI, allowing SMAD7/ubiquitin E3 ligase complexes to exert their inhibitory role towards TβRI and, thus, abolishing TGFβ pro-cytostatic effects [86]. Mutant PTPN3 L232R is frequently found in iCCA and results in loss of its anti-tumorigenic function. This finding could be of clinical importance for the subset of patients that carry *PTPN3* L232R mutations, although a direct correlation between mutant PTPN3 and inactivation of TGFβ signaling in iCCA patients was not addressed in this study.

### 3.3. Noncoding RNAs as Emerging Effectors of TGFβ Signaling during CCA Progression

Noncoding RNA (ncRNA) are transcripts that lack protein-coding potential. Instead, they are transcribed from genes and perform regulatory or structural roles in cells. Noncoding RNA longer than 200 nucleotides are defined as long ncRNA (lncRNA). The rest are collectively designated as short ncRNA and, among many types, include the microRNA (miRNA), whose functional role is to target messenger RNA (mRNA) for degradation or to inhibit mRNA translation, thereby limiting gene expression [87]. The role of ncRNA in cancer progression, downstream of TGFβ signaling, has been appreciated during the last years. For instance, in HCC, the lncRNA activated by TGFβ (lncRNA-ATB) is a target gene of TGFβ signaling and contributes to metastasis by sponging miR-200, thereby stabilizing the EMT transcription factors ZEB1 and ZEB2, and by stabilizing IL11 mRNA, thus favoring tumor cell dissemination [88].

The crosstalk between TGFβ signaling and miRNA in modulating EMT has been investigated in samples from patients with CCA. After measuring the expression levels of epithelial and mesenchymal markers in tumor and adjacent nontumor tissues from 20 patients, Zhang and co-workers observed low expression of the epithelial markers E-cadherin and miR-200b, and high expression of the mesenchymal markers fibronectin and a-smooth muscle actin (α-SMA) in the tumor tissues [89]. In addition, higher levels of TGFβ1 were detected in the tumor tissues, as compared to nontumor adjacent tissues. In vitro experiments using human HCCC and RBE cell lines stimulated with TGFβ confirmed the downregulation of miR-200b and the establishment of an EMT transcriptional program. The mRNA encoding AP2α and MAPK7 proteins were identified as direct targets of miR-200b. Enforced expression of miR-200b led to decreased tumor formation and EMT in an in vivo mouse model, highlighting its tumor-suppressing role and the importance of its repression by TGFβ to elicit the EMT program [89]. In a rat model of iCCA, increased tumor size and enhanced intrahepatic metastasis were detected upon overexpression of TGFβ1 [90]. In addition, TGFβ1 promoted colony formation in a rat bile duct epithelial cell line BDE-Neu. The authors also used RNA interference to deplete TGFB1 and chemical inhibition of TβRI kinase activity by LY2157299 and SB431542 to further confirm that both manipulations negatively affected cell growth and migration of RBE and SSP25 CCA cell lines. At the molecular level, miR-34a was identified as a central effector downstream of TGFβ signaling. The expression of miR-34a was reduced in response to TGFβ, which was followed by an increase in the expression of the direct targets of miR-34a, including CDK6, cyclin D1, and c-Met. Stabilization of CDK6 and cyclin D1 promoted cell growth, and elevated c-Met levels reinforced migration [90]. In another study, the anti-migratory role of miR-34a in CCA was also established, although an alternative mechanism for the miR-34a-mediated suppression of EMT has been described. According to Qiao and co-workers, miR-34a targeted SMAD4 and suppressed TGFβ-induced EMT in vitro. Moreover, the levels of miR-34a and SMAD4 were inversely correlated in human eCCA tissues, with decreased miR-34a and increased SMAD4 expression being observed [91]. The miRNA miR-29a was suppressed by TGFβ in the CCA cell lines FRH–0201 and CCLP–1. In addition, CCA tissues expressed lower miR-29a levels compared to adjacent nontumor tissues. Overexpression of miR-29a dampened the TGFβ-induced cell migration and invasion in CCA cells. The histone deacetylase HDAC4 was identified as a direct target of miR-29a and rescue experiments. Using simultaneous overexpression of both HDAC4 and miR-29a showed a reversion of the EMT phenotype, which was abolished, due to miR-29a overexpression alone. Furthermore, HDAC4 promoted EMT in CCA cells, although the direct molecular targets of HDAC4 were not deeply investigated [92].

A recent report highlighted the importance of intercellular transfer of miRNA through extracellular vesicles (EVs) during CCA progression. As an example, TGFβ represses the expression of miR-30e, a negative regulator of SNAIL in HuCCT1 cells. Enforced expression of miR-30e results in a decreased expression of SNAI1 and of several EMT markers. In addition, miR-30e was packaged in EVs and transported to recipient HuCCT1 cells, where it exhibited its anti-EMT functions. Interestingly, TGFβ treatment reduced the abundance of miR-30e in EVs of CCA cells, suggesting a negative role of miR-30e in regulating EMT under physiological conditions [93].

Although several studies have identified lncRNA associated with CCA progression [94], the knowledge concerning lncRNA acting as regulators of CCA progression in response to TGFβ signaling is currently limited. Nevertheless, the lncRNA TGFβ-induced long noncoding RNA (TLINC) was reported to be highly expressed in response to TGFβ stimulation and to facilitate a pro-inflammatory microenvironment by enhancing cytokines, such as IL8 [95]. This finding strengthens the idea that additional lncRNAs could be effectors of TGFβ-regulated responses in CCA.

More recently, circular RNA (circRNA) emerged in the literature as a new class of ncRNA that may play a critical role in cancer. Numerous circRNA are generated from alternative back-splicing of coding and ncRNA, forming continuous loop without 3′ and 5′ extremities. Recent data highlighted the deregulation of circRNA in several cancers [96]. At the functional level, evidence indicates that circRNA play a key regulatory role at transcriptional and translational levels by possibly acting as miRNA sponges, or as scaffolds for RNA binding proteins (RBP) to form RNA-protein complexes [97]. The singular circular structure of circRNA renders them extremely resistant to exoribonucleases. Therefore, circRNA exibit an expanded half-life, as compared to their linear counterpart RNA. Moreover, studies demonstrated that circRNA may also be found circulating in body fluids, freely or in exosomes [98]. Altogether, these characteristics are promising regarding the capability of circRNA as reliable diagnostic and predictive biomarkers in cancer. Although there is currently no report about circRNA deregulation in CCA, this new class of ncRNA could act as key regulator in the TGFβ network and thereby in cancer. Notably, Goodal et al. demonstrated that TGFβ could promote RNA circularization by inducing the binding of the RBP Quaking on introns flanking circular junctions, which could foster the pro-oncogenic feature of some circRNA [99]. These data reinforce the idea that circRNA could also be effectors of TGFβ-mediated responses in cancer, including CCAs.

### 3.4. Therapeutic Targeting of the TGFβ Signaling in CCA

The importance of the TGFβ signaling in CCA renders this pathway a promising target for developing anti-tumor therapies. Thus, drugs that specifically target the TGFβ pathway at different levels (i.e., maturation of latent TGFβ to active TGFβ dimers, ligand binding to its receptors, TβRI kinase activity) have been or are currently tested for possible anti-tumor effects in different cancers, including liver cancers [100,101,102,103,104]. In CCA, anti-TGFβ-based therapeutic strategies have been mainly evaluated in preclinical models. Thus, in vivo experiments using a rat model of induced liver fibrosis showed decreased levels of fibrosis and CCA in animals treated with a neutralizing monoclonal antibody against TGFβ. In contrast, control animals exhibited extensive liver fibrosis, which, eventually, led to the development of larger tumors [105]. Other mouse xenograft models showed increased tumor dissemination and larger tumors at the site of injection when mice were injected with CCKS-1 cells and simultaneously treated with TGFβ1 as compared to vehicle control-treated mice. Interestingly, the pro-tumorigenic effects of TGFβ were abolished upon administration of a soluble form of TβRII that competes for binding to TGFβ ligands with the membrane-bound TβRII [76]. In addition to these preclinical models, ongoing clinical trials make use of the chimeric antibody M7824, which is composed of the extracellular domain of human TβRII and the C-terminus of human anti-PD-L1 heavy chain [106,107]. M7824 has a double anti-tumor function as it serves as a trap for TGFβ ligand binding at the tumor microenvironment and restricts the immune checkpoint factor programmed cell death ligand-1 (PD-L1), thereby restoring immune responses against the tumor [106,107]. Thus, a recently started multicenter phase II clinical trial is evaluating M7824 monotherapy in locally advanced or metastatic second line biliary tract cancer, including CCAs and gallbladder cancer (NCT03833661).

## 4. Conclusions

**Figure 2:**
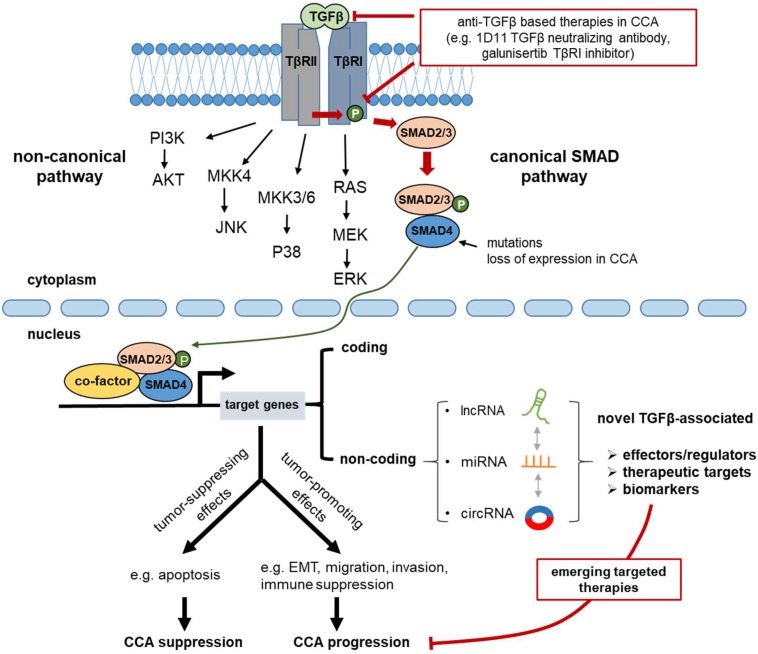
TGFβ signaling in cholangiocarcinoma (CCA) progression. TGFβ activates SMAD-dependent (canonical) and SMAD-independent (non-canonical) pathways in order to evoke transcriptional programs that, ultimately, regulate physiological responses in CCA. Consistent with its dual role in cancer, TGFβ can either prevent CCA progression, by inducing apoptosis, or enhance CCA progression, by promoting EMT, migration, invasion, and suppression of the immune system. Many coding and noncoding TGFβ-target genes can mediate the effects of the pathway during CCA progression and a number of them could potentially serve as drugable targets and biomarkers of CCA. Current therapeutic approaches aim at targeting components of the core pathway, such as the TGFβ ligands and the type I TGFβ receptor (TβRI).

This overview of TGFβ functions in liver homeostasis underlined the critical role of its dysregulation in cancer onset and progression. The fact that CCA evolves in a desmoplastic microenvironment, whereby TGFβ is extremely abundant and frequently associated with a poor prognosis, emphasizes the clinical relevance of TGFβ-targeted-therapies. Unfortunately, the literature described TGFβ as an elusive target considering that, depending on the cell transcriptional context, it exhibits a dichotomous action. Thus, any therapeutic strategy aiming at modulating the TGFβ pathway must consider its potential repercussion, either by repressing the pro-apoptotic and tumor suppressor arm, or by improving the pro-oncogenic and pro-metastatic action. A better insight into the molecular mechanisms regulating the functional duality of TGFβ could improve the efficiency of targeted therapies for a better management of patients with CCA (Figure 2).

The latest evidence promises a bright future for ncRNAs including miRNA, lncRNA, and circRNA as innovative downstream TGFβ effectors, or as clinically relevant biomarkers in CCA (Figure 2). A better understanding of the intricate and coordinated network of coding and ncRNAs will certainly allow us to elucidate how the functional duality of TGFβ in CCA is regulated. Some of these ncRNAs are also found circulating in body fluids and could represent specific biomarkers for a better management of patients with CCA.
